# Selecting Strategies to Reduce High-Risk Unsafe Work Behaviors Using the Safety Behavior Sampling Technique and Bayesian Network Analysis 

**Published:** 2017-03-04

**Authors:** Fakhradin Ghasemi, Omid Kalatpour, Abbas Moghimbeigi, Iraj Mohammadfam

**Affiliations:** ^1^ Center of Excellence for Occupational Health, Research Center for Health science, School of Public Health, Hamadan University of Medical Sciences, Hamadan, Iran; ^2^ Modeling of Non communicable Diseases Research Center and Department of Biostatistics, School of Public Health, Hamadan University of Medical Sciences, Hamadan, Iran

**Keywords:** Behavior, Accident Prevention, Safety Management, Construction Industry, Data Mining, Occupational Injuries

## Abstract

**Background:** High-risk unsafe behaviors (HRUBs) have been known as the main cause of occupational accidents. Considering the financial and societal costs of accidents and the limitations of available resources, there is an urgent need for managing unsafe behaviors at workplaces. The aim of the present study was to find strategies for decreasing the rate of HRUBs using an integrated approach of safety behavior sampling technique and Bayesian networks analysis.

**Study design:** A cross-sectional study.

**Methods:** The Bayesian network was constructed using a focus group approach. The required data was collected using the safety behavior sampling, and the parameters of the network were estimated using Expectation-Maximization algorithm. Using sensitivity analysis and belief updating, it was determined that which factors had the highest influences on unsafe behavior.

**Results:** Based on BN analyses, safety training was the most important factor influencing employees' behavior at the workplace. High quality safety training courses can reduce the rate of HRUBs about 10%. Moreover, the rate of HRUBs increased by decreasing the age of employees. The rate of HRUBs was higher in the afternoon and last days of a week.

**Conclusions:** Among the investigated variables, training was the most important factor affecting safety behavior of employees. By holding high quality safety training courses, companies would be able to reduce the rate of HRUBs significantly.

## Introduction

Although construction safety has been significantly improved in recent years, accidents continue to occur and construction still is one of the most risk-posing industries in the world^1, 2^. In the developing countries like Iran, there are many deficiencies that create an even worse situation, some of these deficiencies are as follows: the lack of rules and regulation, inadequate and incomplete government inspection, unskilled workers migrated from other places and from even other countries, employed for just a short period of time, higher pressure in terms of time and economics, absence of a comprehensive accident recording and reporting system,^3, 4^.

It is quite clear that many factors contribute to construction accidents. Furthermore, managing safety at construction sites and preventing construction accidents is a complex issue^3^. One factor, which can be traced in most accidents, is human element in the shape of an unsafe behavior or a human error. The role of human element in occupational accidents has been addressed in many studies. Eighty five percent of accidents are caused by unsafe acts^5^.Unsafe behavior has been recognized as a main factor in at least 70% of construction accidents ^6^. All construction accidents have three roots; failing to identify an unsafe condition, continuing a work activity, regardless of its identified unsafe condition, and taking deliberately an unsafe behavior; all these three reported roots are related to human factors ^7^. Workers or work team is the most important key factor involving in construction accidents^8^. Human behavior has been introduced as one the most important factors in road collision, as well^9^.

The importance of human behavior in construction safety has motivated both researchers and practitioners to focus on behavioral based safety programs^10-12^. In implementing a behavioral based safety program, selecting intervention strategies is of vital importance.

Bayesian networks are a useful tool attracted much attention in recent years, particularly in the field of occupational safety and risk assessment. For example, Khakzad et al. ^13, 14^ introduced it as a strong tool for conducting risk analysis in process industries. Leu and Chang ^15^ employed it to assess the risk of steel construction project. Martín et al.^16^ utilized it to analyze accidents resulting from falls from heights, and Zhao et al. ^17^ used it to assess factors affecting the hazardous material transportation accidents. Previous studies, have utilized the technique for selecting strategies to improve safety behavior at workplaces ^18, 19^; however, none of them have used safety behavior sampling (SBS) technique, a valid and reliable method for observing and assessing the real behavior of employees at workplace^20^, as a basis for training their networks.

In the present study, we aimed to utilize an integration approach of BN analysis and SBS technique to find improvement strategies.

## Methods

The present study was conducted in a large power plant construction site in Iran. The study was composed of five main steps as follows:

**Step 1:** Defining HRUBs and conducting SBS

The SBS is one the most used techniques for behavior sampling^20, 21^. SBS is based on the approximation of the binomial distribution by normal distribution function in the situation that n (number of observations) is large and p (probability of success) is close to 0.5 ^22^. For achieving this purpose, after providing a list of possible unsafe behaviors and training the sampler people, a pilot study must be conducted. The aim of the pilot study is to estimate parameter p by equation 1, in which N0 is the total number of observations and N1 is the number of the desired observations.

(Eq. 1) p=N1N0

Since the value of p is determined, the total number of required observations is calculated from equation 2.

**Figure:**
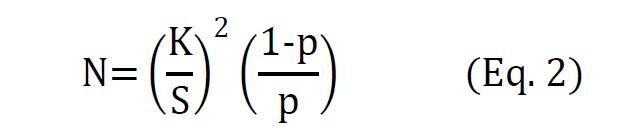

In equation 2, K is equal to Z1-α which should be read from the standard normal distribution table and S is the desired amount of accuracy. In the present study, the level of confidence (α) was 0.95, consequently, K was approximately equal to 2, and S was equal to 0.05. These values of K and S have commonly been adopted in studies in which this technique has been used^20, 21^.

During the sampling process, employees should be unaware of being observed. Moreover, the sampling should be done in a random manner and cover all the hours of a day and all the days of a week.

Observers observed employees' behavior in a random manner and record the name of employees and the type of his behavior. A trained and experienced safety officer made all observations during almost five weeks. Front line employees were the focus of the study, so all observations were conducted on them. As the focus of the present study was on the HRUBs, only those behaviors that were able to cause immediate serious injuries were regarded as HRUBs. Accordingly, such unsafe behaviors as those related to ergonomics issues (awkward posture, carrying a heavy load) and those related to general PPEs were not regarded as HRUB. Some examples of HRUBs are presented below:

● Unsafe behavior related to construction equipment;
- Removing the safe guards of grinders (stationary or portable) while handling it
- Using unsafe welding equipment
- Using machines such as grinders for an irregular purpose
● Unsafe behavior related to cranes and heavy trucks;
- Using improper or defected slings
- Improper rigging
- Rapid swinging of suspended loads
- Unsafe work practices in the vicinity power lines
- Driving heavy trucks too fast
- Driving heavy trucks out of a predetermined area
● Unsafe behavior related to performing a task at height
- Ignoring to use safety harness
- Failure to anchor safety harness to a reliable point
- Walking on an unsafe surface/platform at height
- Improper use of a ladder
- Horseplay at height

**Step 2:** Extracting information from employees' records

The records of employees were reviewed and some variables supposed to affect their behavior at the workplace were extracted. Accordingly, six variables were selected including age, experience, marital status, education level, number of training courses that employees participated and previous accidents. In addition, time of day and day of week were also two other variables recorded by the observers when the SBS technique was conducting. After that, each variable was discretized in distinct states (Table 1).

**Table 1:** Variables of the study and their states

**Variables**	**States of the variable**
Age	State 1: "under 30 years" for ages lower than 30 years State 2: "from 30 to 40 years " for ages between 30-40 years State 3: "above 40 years " for ages higher than 40 years
Experience	State 1: "under 1 years " for experience lower than 1 years State 2: "from 1 to 5 years " for experience between 1 and 5 years State 3:"above 5 years " for experience between 5 and 10 years
Marital status	State 1: "yes" for a married employee State 2: "no" for an unmarried employee
Previous accident	State 1: "no accident" for those cases without previous accident State 2: "minor accident" for those cases with a previous accident that has not resulted in a lost working day State 3: "major accident" for those cases with an accident that has resulted in at least one lost working day
Educational level	State 1: "primary" for an employee with primary education level State 2: "high school" for an employee with high school education level State 3: "academic" for an employee with academic education level
Weekday	State 1: "first days" for two first days of a week State 2: "middle days" for three days midweek State 3: "last days" for two last days of a week
Daytime	State1: "from 8am to 11am" for observations that have been made in time interval from 8 am to 11 am. State 2: "from 11am to 2pm" for observations that have been made in time interval from 11 am to 2 pm. State 3: "from 2pm to 6pm" for observations that have been made in time interval from 2 pm to 6 pm.
Training	State 1: "s1" for employees who have participated in one or two safety training courses, State 2: "s2" for employees who have participated in three or four safety training courses, State 3: "s3" for employees who have participated in five or more safety training courses,
HRUB	State 1: "no" for a safe behavior State 2: "yes" for HRUBs

**Step 3:** Constructing the BN

A Bayesian network is a graphical model composed of a set of nodes, directed arcs, and conditional probability tables. Nodes represent random variables, directed arcs determine the causal relationship between variables, and CPTs demonstrate the probability of various states of a node according to different configurations of its parent states. Each BN can be represented by ordered pairs N=(G,p), in which G(V,E) is a directed acyclic graph (DAG) with a set of nodes V = {X_1_,X_2_,X_3_,…,X_n_} and a set of directed edges E, and p is the joint probability over the variables V which is calculated using equation 3 ^23^. Moreover, Bayesian network has been regarded as a simple and strong classifier ^23^.

(Eq. 3) $$P(X_{1},X_{2},X_{3},...,X_{n})=\prod_{i=1}^{n}P(X_{i}|Parent(X_{i}))$$

After determining the variables and discretizing them, the causal relationships among them should be assigned. There are two ways using which such relationships can be constructed; in the first approach, the Bayesian network is constructed using domain knowledge experts, and in the second approach, various structure-learning algorithms are employed for constructing such a network. Commonly, the former approach is used by researchers, in the sense that the use of learning algorithms for structuring a Bayesian network has some disadvantages. For example, using such algorithms generally needs a large data set and the final Bayesian network structure may make a causal link between some variables that is not matched with the expert knowledge. In practice, we often have a limited data set; consequently structuring BN using domain expert knowledge is more popular. The use of experts' knowledge for constructing a Bayesian network has been used earlier ^16, 17, 19^. There are several ways for soliciting expert knowledge, including focus group and Dempster-Shafer theory. We used a focus group approach.

**Step 4:** Parameter estimation;

The fourth step of the framework is to compute conditional probability tables (parameters) of each variable. These tables for all variables can be computed using Expectation-Maximization (EM) algorithm based on the dataset provided in the previous step. The algorithm is a two-step process, repeated for a finite number of times until a convergence is obtained. The step 1 is called expectation; where the data set is completed by assigning an initial probability distribution for each parameter, and then estimating missing values according to the assigned initial probability distribution. The step 2 is called maximization; in this step, the completed data set is utilized to estimate a maximum likelihood for each parameter^23^. Commercially available software Netica developed by Norsys, used in this study, is equipped with this algorithm.

**Step 5:** Selecting intervention strategies

BNs enable us to anticipate the effects of changes in some variables on other variables. This feature can be used for choosing intervention strategies^19, 20^. The basis of belief updating is the Bayes theorem (equation 4), which guides us to modify our belief regarding the probability of an event after observing some evidences ^24^. In fact, most BN-based analyses are based on this feature.

(Eq. 4) $$P(A|B)=\frac{P(B|A).P(A)}{P(B)}$$

Finally, sensitivity analysis was conducted. Sensitivity analysis would enable us to rank the variables by the magnitude of their effects on unsafe behavior. As explained by García-Herrero et al.^25^, belief updating can be used in this regard. Equation 5 was used to perform this step of the study.

**Figure:**
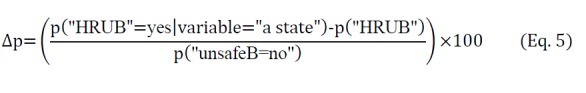


## Results

Two hundred observations were made, out of them 90 cases were regarded as a HRUB. Therefore, according to equation 3, about 1960 observations were done. These observations have been made during the following five weeks.

**Figure 1:**
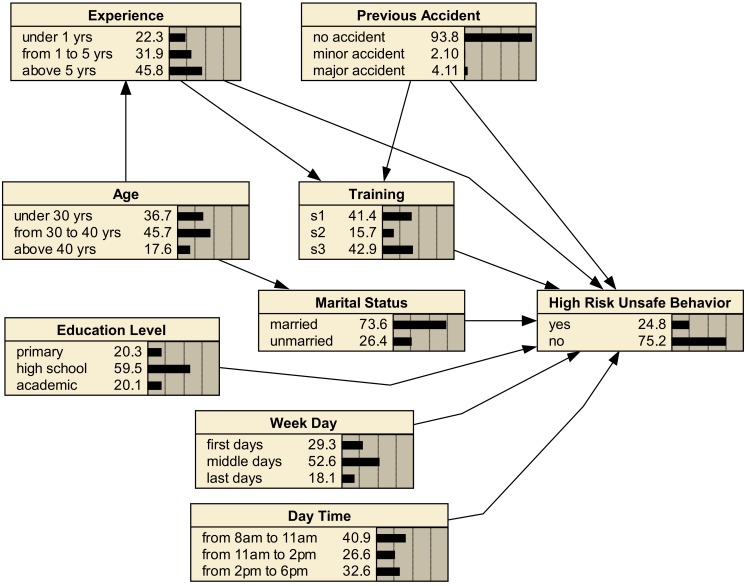
Prior probability of variables after computing the parameters of the network

Using the experts’ knowledge (a focus group containing three safety experts) the Bayesian network depicted in Figure 1 was elicited. According to this network, almost all variables directly affect behavior, except variable "age" which its effect is mediated by experience and marital status. In this network, we connected "previous accident" node to "training" node because the company obliges employees with accident to participate in more safety training courses.

After constructing the desired Bayesian network, its parameters should be estimated. The SBS technique and E-M algorithm were used to accomplish this purpose. Using the SBS the required dataset was provided and using the E-M algorithm, the parameters (CPTs) of the network were computed. After this step, the network depicted in Figure 1 was resulted. This figure represents the marginal probabilities calculated from the CPT of each variable.

An interesting feature of the Bayesian network depicted in Figure 1 is its applicability in explaining the present situation of the workplace in terms of the modeled variables. For example, 24.8% of observed behaviors were HRUBs, 4.11% of employees had a previous major accident, 73.6% were married, most of them aged between 30-40 yr, and had an experience above 5 yr, almost half of the observations were done in the middle days of a week, and most employees (59.5%) had a high school degree.

**Figure 2:**
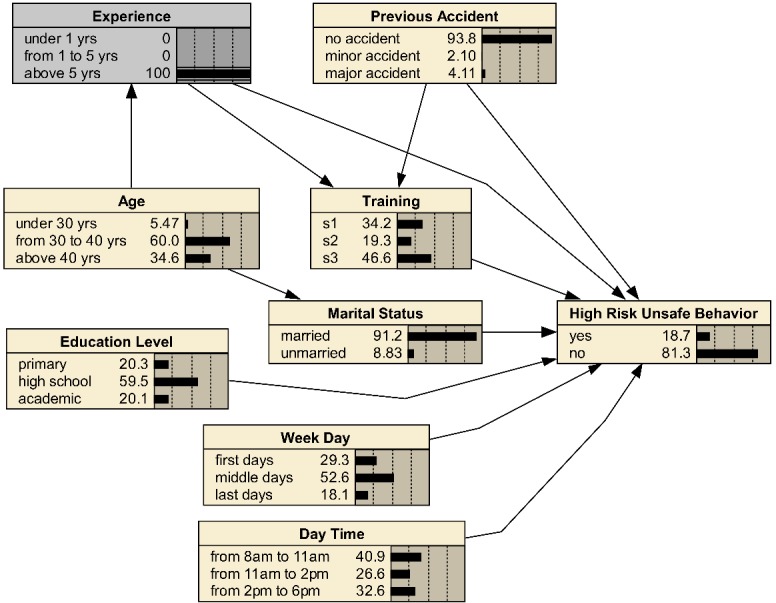
Introducing evidence on "experience node" and postrior probability of other variables

In the next step, the network was analyzed in order for finding the best strategies for behavior improvement. All the analyses were performed based on a feature of BNs known as "belief updating" using new evidences. The feature is based on Bayes theorem and fully explained by Nielsen and Jensen ^23^. The main application of this feature is to evaluate the effect of changes in some variables on other variables. An example of belief updating is demonstrated in Figure 2, in which the evidence has been introduced to the "experience" node. Comparing this figure and Figure 1, we can see how the probability of various states of other variables is changed.

**Figure 3:**
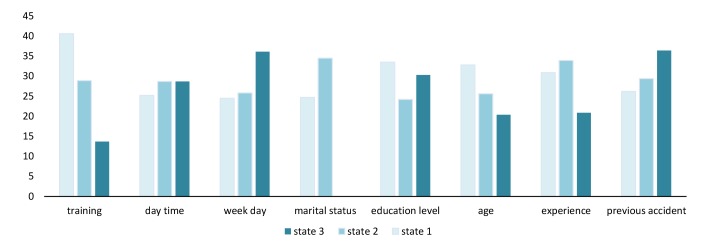
Probability of High-risk unsafe behaviors at various states of other variables

Using this procedure, the probability of HRUBs corresponding to states of other variables was calculated and shown in Figure 3. Accordingly, the lowest probability of HRUBs was associated with <training=s3).

Moreover, the results of sensitivity analysis performed using the Bayesian networks are displayed in Table 2. The rightmost column of this table represents the rank of the variables by the magnitude of their effects on safety behavior. As we see in this column, training and experience were variables with the highest effects on safety behavior. In addition to equation 5, another way to perform a sensitivity analysis is to use mutual information. As we can see in this table, the results of both sensitivity analysis approaches are the same.

**Table 2:** Sensitivity of HRUBs to changes of other variables

**Variables**	**States**			**Absolute mean** **of variations**	**Mutual information**	**Ranking**
	**State 1**	**State 2**	**State 3**			
Previous Accident	-4.03	7.69	33.33	15.02	0.00597	6
Experience	13.19	24.18	-23.44	20.27	0.01270	2
Age (yr)	20.15	-6.23	-25.27	17.22	0.00764	4
Education Level	22.71	-11.36	10.99	15.02	0.00570	7
Marital Status	-9.52	26.37	-	17.95	0.00674	3
Week Day	-10.26	-5.49	32.23	16.00	0.00597	5
Day Time	-7.69	5.13	5.13	5.98	0.00061	8
Training	48.72	5.86	-49.82	34.80	0.05451	1

## Discussions

Unsafe behaviors have always been a challenging area in managing safety and health in workplaces, especially in construction sites. In the present study, we used Bayesian networks approach to show how some personal and temporal factors affect employees' behavior in a construction site and to find strategies for improving high-risk unsafe behavior. Bayesian networks are a powerful tool for graphically analyzing a desired part of the world according to the causal relationship between them and has been used for finding intervention strategies by several studies ^18, 19^. Moreover, the safety behavior sampling was used to collect the required data set. The network was built using the focus group approach, and its parameters were estimated using E-M algorithm.

The results of the present study revealed that the rate of HRUBs in the workplace was 24.8%, which is comparable with another study^20^.

Among the investigated variables, safety training was the most important factor affecting the rate of HRUBs at the workplace. Moreover, if we try to hold more safety training courses, the rate of HRUBs can be reduced to as low as 12.3%. The importance of safety training has been stressed ^26^. Lack of safety awareness is one the most important factors causing employees to engage in unsafe behavior^27^. Training plays a major role in enhancing employees' knowledge and awareness about the hazards of their workplace. Safety training can improve personal safety attitude and thereby safety behavior^28^.

In the present study, the effect of other factors such as age, experience, marital status, previous accident, time of day, and day of week were also investigated. However, their effects were not as high as to be considered significant, while some studies have reported a significant correlation between such factors and the rate of occupational accidents. For example, Ling et al. ^29^ stated that the rate of accidents was higher at the times near the rest breaks, whereas, in the present study, we did not observe a sharp increase in the rate of HRUBs during the hours between 11 am and 2 pm (Figure 3). In the same vein, most fatal injuries at construction sites tend to occur in the afternoon^30^, which is in line with our findings, because we observed that the probability of HRUBs increased after 2 pm. Age, time of the accident, and weekday are among factors influencing the severity of accidents at construction sites^31^. The same results were reported by Lopez Arquillos et al ^32^. Moreover, young workers are more prone to accidents than do older counterparts^33, 34^. This finding is totally consistent with our findings, because as depicted in Figure 3, the rate of HRUBs increases as the age of employees decreases. Furthermore, Amiri et al. ^35^ conducted a meta-analysis study in this area, which showed the risk of occupational accidents is higher during weekends and among young workers, which also is in line with the results of the present study. The rate of HRUBs was much lower among married employees (22.3% vs. 32%), which is in line with our previous study ^36^ and indicates that social support was very important in shaping employees' behavior at workplaces.

Furthermore, safety training was the most important factor affecting HRUBs and any attempt to improve employees' behavior should start by concentrating on this variable.

Lastly, in the present study, BN and SBS were utilized in integration to find strategies for reducing the rate of HRUBs at workplaces. In comparison with other techniques normally used to investigate unsafe behavior such as path analysis (PA) and structural equation modeling (SEM), the BN has the advantage of being capable to predict the intended outcome, while PA and SEM are very powerful tools in explanation of behavior and normally are not used for prediction ^37^.

Moreover, the present study had several limitations, which should be covered by future studies.

Although using SBS technique for assessing safety behavior has several advantages (in contrary to questionnaire based methods which always are in danger of conservative responses from employees, using this technique, we would observe and record the real behavior of employees that ensures their correctness, another benefit of SBS is that it provides a large database which can be used for training the network.), it suffers from several drawbacks as well; the main problem associated with this technique is that we cannot record so many variables while using this technique. It is recommended for future studies to conduct an interview or a questionnaire study after the SBS is completed, on the same employees whom behaviors were observed to include more variables into the network.

## Conclusions

Training was the most important factor affecting safety behavior of employees. By holding high quality safety training courses, companies would be able to reduce the rate of high-risk unsafe behavior significantly.

## Acknowledgements

The authors would like to thank Hamadan University of Medical Sciences for the financial support under PhD thesis scheme (grant number: 9411276661).

## Conflict of interest statement

The authors declare no conflict of interest.

## Funding

Hamadan University of Medical Sciences

## Highlights

- Safety behavior sampling was used to provide a data base of cases,
- Bayesian network was utilized to analyze high risk unsafe behavior,
- Safety training was the most influencing factor,
- The rate of high risk unsafe behavior is higher in the afternoon and last days of a week
- The rate of high risk unsafe behavior increases by decreasing the age of employees
